# The effect of hookah use on COVID-19 related adverse outcomes: Lessons learned from integrating STEPs 2016 and national COVID-19 registration databases

**DOI:** 10.18332/tid/144052

**Published:** 2022-01-28

**Authors:** Azin Ghamari, Negar Rezaei, Mansour Ranjbar, Farshad Farzadfar

**Affiliations:** 1 Non-Communicable Diseases Research Center, Endocrinology and Metabolism Population Sciences Institute, Tehran University of Medical Sciences, Tehran, Iran; 2 Endocrinology and Metabolism Research Center, Endocrinology and Metabolism Clinical Sciences Institute, Tehran University of Medical Sciences, Tehran, Iran; 3 Non-communicable Diseases and Mental Health Unit Head, World Health Organization, Tehran, Iran

**Keywords:** smoking, hookah, ICU admission, ventilation therapy, COVID-19

## Abstract

**INTRODUCTION:**

Amidst the COVID-19 pandemic, an international effort has been concerted to identify the factors associated with more adverse outcomes to better allocate resources and perform more effective targeted preventive measures. This study aims to describe the risk of COVID-19 adverse outcomes among individuals with a history of being ever cigarette smokers and being ever hookah users.

**METHODS:**

We combined two databases, including the Iran national registry of COVID-19 patients, including 2020 with 656258 hospitalized patients and STEPs survey 2016 with 30541 participants. After merging the two databases using the national ID, the association was investigated between being ever smoker or hookah user and the adverse outcomes of COVID-19 including death, need for a ventilation therapy, and admission in the intensive care unit (ICU), among 474 severe acute respiratory infections (SARI) cases and 211 PCR-positive patients.

**RESULTS:**

Among 211 PCR-positive patients, 40 (19%) patients were ever hookah users and 28 (13.3%) were ever cigarette smokers. Death occurred in 27 (12.8%) patients and severe COVID-19 in 17 (11.6%). Among 211 PCR-positive patients, ever cigarette smokers had 4.2 times (95% CI: 1.1–15.4, p=0.03) higher odds of ICU admission and 4.2 times (95% CI: 1.1–15.4, p=0.03) increased odds for need of ventilation, compared with non-smokers. Besides, ever hookah users had 3.9 times (95% CI: 1.1–13.6, p=0.03) higher odds for need of ventilation therapy, compared with non-hookah users.

**CONCLUSIONS:**

Tobacco use and hookah smoking were associated with adverse outcomes among COVID-19 patients in Iran.

## INTRODUCTION

The novel coronavirus disease 2019 (COVID-19) has become a challenge for all countries across the globe. Amidst the COVID-19 pandemic, an international effort has been concerted to identify the factors associated with more adverse outcomes to better allocate resources and perform more effective targeted preventive measures. One important factor is known to be smoking and hookah use^1,2^. Generally, it has been shown that smoking and hookah use increases the risk of respiratory system infection due to several reasons, including cardiopulmonary comorbidities associated with smoking, impaired mucociliary system, altered immune system, and different healthcare seeking behavior^3^. Additionally, the risk of COVID-19 infection increases among hookah users due to social gathering in crowded places and having close contact with each other, sharing the same pipes, difficult-to-clean pipes and cold water reservoirs^4^. Consequently, due to the increased viral load, increased COVID-19 severity and adverse outcomes could be anticipated. In this regard, this study aims to describe the risk of COVID-19 adverse outcomes among individuals with a history of being ever cigarette smokers and being ever hookah users. To the best of our knowledge, no studies have evaluated the association between hookah use and COVID-19 adverse outcomes in Iran.

## METHODS

We combined two databases, including the Iran national registry of COVID-19 patients and STEPs survey 2016. STEPs survey 2016 was performed as a large-scale cross-sectional study for the surveillance of risk factors of non-communicable diseases in Iran, including data regarding being ever smoker and hookah user among a total number of 30541 participants^5,6^. The prevalence of being ever smoker and ever hookah user in the STEPs survey 2016 was 4363 (14.3%) and 6326 (20.7%), respectively. In the COVID-19 registry system (MOH portal), from the beginning of pandemic in Iran until November 2020, 656258 hospitalized patients were identified as SARI (Severe Acute Respiratory Infection) and 270959 as PCR positive for COVID-19. After merging the two databases using the national ID, the association was investigated between being ever smoker or hookah user and the adverse outcomes of COVID-19 (including death, need for a ventilation therapy, and admission in the intensive care unit), among 474 SARI-cases and 211 PCR-positive patients.

### Statistical analysis

All data were analyzed using the STATA version 12. Descriptive results are presented as mean ± standard deviation or frequency (percentage). The association between being ever smoker/hookah user and COVID-19 adverse outcomes was reported by crude odds ratios (ORs) and 95% confidence intervals. A p<0.05 was considered statistically significant.

## RESULTS

Among 474 SARI patients, 97 (20.7%) were ever hookah users and 72 (15.4%) were ever cigarette smokers. Death occurred in 58 (12.2%) patients and severe COVID-19 in 48 (15.3%) (Table 1). Among 211 PCR-positive patients, 40 (19%) patients were ever hookah users and 28 (13.3%) were ever cigarette smokers. Death occurred in 27 (12.8%) patients and severe COVID-19 in 17 (11.6%) (Table 1). Among 211 PCR-positive patients, ever cigarette smokers had 4.2 times (95% CI: 1.1–15.4, p=0.03) higher odds of ICU admission and 4.2 times (95% CI: 1.1–15.4, p=0.03) increased odds for need of ventilation, compared with non-smokers (Table 2). Besides, ever hookah users had 3.9 times (95% CI: 1.1–13.6, p=0.03) higher odds for need of ventilation therapy, compared with non-hookah users (Table 2). It is noteworthy that all ever cigarette smokers who were admitted to the ICU needed ventilation therapy.

**Table 1:** Demographic data of patients hospitalized for COVID-19 and COVID-19 outcomes

*Variable*	*Patients with SARI ^a^ (n=474) n (%)*	*PCR positive patients (n=211) n (%)*
**Age** (years), mean ± SD	62.63 ± 16.68	61.27 ± 16.04
<20	1 (0.21)	0 (0)
20–40	50 (10.55)	21 (9.95)
40–60	132 (27.85)	70 (33.18)
>60	291 (61.39)	120 (56.87)
**Gender**, Male	252 (53.16)	120 (56.87)
**Ever hookah use^b^**	97 (20.68)	40 (18.96)
**Ever smoking^c^**	72 (15.35)	28 (13.27)
**Outcome**		
Death	58 (12.24)	27 (12.80)
Recovery^d^	332 (70.04)	156 (73.93)
Under treatment	84 (17.72)	28 (13.27)
Sever condition^e^	48 (15.29)	17 (11.64)

a SARI: acute respiratory illness in addition to fever or measured fever ≥38°C and cough, occurred within the past 10 days plus need for hospitalization.

b Ever hookah use: positive history of ever smoking hookah, even one or two puffs.

c Ever smoking: being ever cigar/cigarette smoker.

d Recovery: being clinically stable and COVID-19 therapy discontinuation 14 days after being symptomatic or positive PCR test.

e Severe condition: being not dead and ICU admitted with or without need for ventilation therapy.

**Table 2:** The association between being ever smoker, being ever hookah user and COVID-19 adverse outcomes

*Outcome*	*Variables*	*Patients with SARI ^a^ (n=474)*		*PCR positive patients (n=211)*	
		*OR (95% CI)*	*p*	*OR (95% CI)*	*p*
Mortality	Ever smoking^b^	1.01 (0.47–2.16)	0.97	0.79 (0.22–2.84)	0.72
	Ever hookah use^c^	1.40 (0.74–2.64)	0.30	1.60 (0.62–4.1)	0.33
ICU admission	Ever smoking	1.88 (0.84–4.18)	0.12	4.19 (1.14–15.38)	**0.03**
	Ever hookah use	1.70 (0.80–3.60)	0.16	2.60 (0.72–9.36)	0.14
Ventilator therapy	Ever smoking	1.75 (0.72–4.25)	0.21	4.19 (1.14–15.38)	**0.03**
	Ever hookah use	2.02 (0.91–4.48)	0.08	3.93 (1.13–13.6)	**0.03**

a SARI: acute respiratory illness on addition to fever or measured fever ≥38°C and cough, occurred within the past 10 days plus needs for hospitalization;

b Ever smoking: being ever cigar/cigarette smoker.

c Ever hookah use: positive history of ever smoking hookah, even one or two puffs.

## DISCUSSION

To the best of our knowledge, this is the first study performed in Iran describing the risk of COVID-19 adverse outcomes among ever cigarette smokers and ever hookah users; compared with non-smokers, ever cigarette smokers had 4.2 times higher odds of ICU admission and need of ventilation. Furthermore, compared with non-hookah users ever hookah users had 3.9 higher odds for need of ventilation therapy.

The prevalence of hookah use is increasing worldwide; 2.6 million people use it in the US^7^ and its usage is increasing among young individuals in Canada^8^. Besides the augmenting prevalence of its usage in western countries, hookah use is more popular in India, the Arab countries, Kenya, South Africa, Turkey, and Iran^9^. Its usage is a socially established phenomenon in Iran; many people use it in their home, in restaurants and in traditional cafés; 24% of Iranian males and 11.3% of Iranian females used it according to a cross-sectional study performed by Hessami et al.^10^ in 2016. Its prevalence is probably higher, as being ever hookah user was not taken into account, in addition to the possible occurrence of under-reporting bias. Another study reported the rate of ever tobacco use, ever daily cigarette smoking, current tobacco use, and current daily cigarette smoking to be 21.1%, 14.6%, 14.2% and 10.1%, respectively, among both males and females^11^. Before the COVID-19 era, the Iranian healthcare system and policymakers were endeavoring to hinder its consumption^11,12^. Although being associated with many unfavorable outcomes and becoming a global health crisis, the COVID-19 pandemic highlighted the negative impact of hookah use and smoking on infection trajectory, which could be considered an opportunity to precipitate in hookah cessation. The findings of the current study indicate that being an ever smoker increased the odds of ICU admission and need of ventilation therapy by approximately 4 times, and being ever hookah user increases the risk for need of ventilation by 3.9 times. Another study, performed by Kalan et al.^13^ in Iran, reported that among a total sample of 193 patients, 7.8% and 7.3% had a history of hookah use and smoking within one month prior to hospitalization, respectively; 6.7% of hookah users were admitted to ICU and 20% died. Two scientific briefs were provided by World Health Organization (WHO) in 2020, indicating that there is a significant association between smoking and hookah use and COVID-19 severity and risk of death^2,4^. Many countries took the occasion to impose restrictions against hookah use. Fifteen countries in the Eastern Mediterranean Region had provisionally restricted hookah use in both indoor and outdoor places^14^. Its usage has already been forbidden in Cairo, Egypt, and Abu-Dhabi and Dubai, in the United Arab Emirates^9^. Also, hookah use in public places has been banned in Ontario, Alberta and other provinces in Canada^9^.

### Limitations

This study has several limitations. First, it had a small sample size, performing another study with larger sample size is recommended. Second, it lacked individual or biochemical information. Third, heterogeneity of data collection exists in the databases used.

## CONCLUSIONS

Given its association with COVID-19 adverse outcomes, in addition to WHO recommendations for restricting hookah use and cigarette smoking during the COVID-19 pandemic^2,4^, it is an opportunity to restrict hookah use in Iran, as it lowers the COVID-19 burden. Additionally, it is a good moment to implement preventive strategies for its control in the future^11,15^.

## Data Availability

The data supporting this research cannot be made available for privacy or other reasons.
